# 气管、支气管腺样囊性癌的诊断与治疗

**DOI:** 10.3779/j.issn.1009-3419.2010.06.016

**Published:** 2010-06-20

**Authors:** 明 秦, 瑜 傅, 大平 于, 绍发 许, 鸣 韩, 子彤 王

**Affiliations:** 101149 北京，北京结核病胸部肿瘤研究所暨北京胸科医院胸外科 Department of Thoracic Surgery, Beijing Research Institute of Tuberculosis and Thoracic Tumor, Beijing Chest Hospital, Beijing 101149, China

**Keywords:** 腺样囊性癌, 治疗, 预后, Adenoid cystic carcinoma, Treatment, Prognosis

## Abstract

**背景与目的:**

腺样囊性癌是一种原发于支气管肺部的低度恶性肿瘤。本研究回顾性分析本院1958年1月-2007年12月共50年间收治的43例患者，探讨原发性气管、支气管腺样囊性癌的临床特点、病理特征和治疗方法。

**方法:**

总结我院50年间收治的43例原发性气管、支气管腺样囊性癌患者，其中40例采取手术治疗，3例患者在纤支镜下行介入治疗（均经手术或病理证实）。全组43例患者术后均无并发症发生，无手术死亡，总随访率为97.6%（42/43），1例失访按死亡计。

**结果:**

本组43例患者中，3年生存率为100%（41/41），5年生存率为89.5%（34/38），10年生存率为87.1%（27/31）。

**结论:**

原发性气管、支气管腺样囊性癌是一种少见的低度恶性肿瘤，临床症状不典型，早期发现并采取手术联合放疗是最好的治疗手段，未手术者在内镜下介入治疗也是一种较好的治疗方法。

## Abstract (en)

**Background and objective:**

Adenoid cystic carcinoma is primary bronchopulmonary carcinoma with low malignancy, and 43 patients treated in the past 50 years in our hospital were retrospectively studied. The aim of this study is to discuss the clinical symptoms, pathologic characteristic and therapeutic method of primary tracheal or bronchuotracheal adenoid cystic carcinoma.

**Methods:**

This study summarized total 43 patients of primary tracheal or bronchus adenoid cystic carcinoma treated in our hospital from Jan. 1958 to Dec. 2007. Among them, 40 patients were treated by surgical resection, and 3 patients were treated by fiberoptic bronchoscope's interventional treatment.

**Results:**

The 1-yr, 3-yr, 5-yr survival rates of the 43 patients above were 100% (41/41), 89.5% (34/38), 87.1% (27/31), respectively.

**Conclusion:**

Primary tracheal or bronchus adenoid cystic carcinoma are rare and low malignancy carcinoma. The clinical symptoms of them are not typical. The best treatment is early detection and taking measures of operation plus radiotherapy. The other palliative treatment is fiberoptic bronchoscope's interventional treatment.

气管腺样囊性癌（tracheal adenoid cystic carcinoma）又称圆柱瘤，是一种少见的原发于气管、支气管的低度恶性肿瘤，临床易造成误诊而延误治疗^[1]^，手术配合放疗效果好^[2]^，总结我院1958年1月-2007年12月50年间收治的43例气管、支气管腺样囊性癌患者，将诊断、治疗效果及治疗经验分析如下。

## 1 资料与方法

总结我院50年间收治的43例原发性气管、支气管腺样囊性癌患者，其中男性23例，女性20例，男女比例为1.15：1，占我们同期收治的支气管肺部肿瘤的0.95%，在同期收治的支气管低度恶性肿瘤中占18.3%。年龄19岁-65岁，平均43岁。病程2周-4年，多为3个月。主要症状与体征：刺激性咳嗽22例，其中7例伴痰血，11例患者主要以胸闷、气短为主诉症状，其中5例伴发热，6例患者以喘息伴吸气性哮鸣音为主要症状（其中2例按喘息性支气管炎在呼吸内科治疗近3个月后症状仍无缓解而来我院胸外科就诊），另有3例因体检发现肺部阴影而就诊。

胸部X线及CT检查发现病变主要为边缘规则、密度均匀的阴影，增强扫描病灶有不同程度强化。中心型40例，其中6例伴肺不张，与中心型肺癌较难鉴别；周围型3例。行纤维支气管镜检查31例，其中30例发现气管、支气管腔内呈结节或菜花样突出的肿物，1例未发现异常。术前取活检经病理证实为腺样囊性癌者19例（图 1）。

**1:**
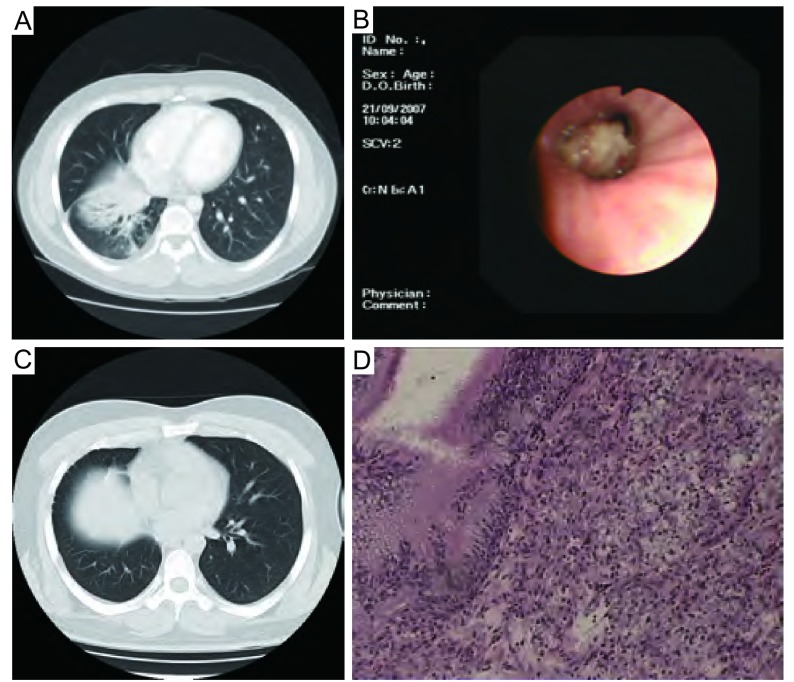
患者诊断相关图片。A：术前CT；B：术前纤支镜；C：术后CT；D：术后病理 One patient's pictures related to diagnosis. A: Preoperative CT; B: Preoperative bronchofiberscope; C: Postoperative CT; D: Postoperative pathology

所有患者中40例采取手术治疗，其中全肺切除并隆突楔形切除成形术5例（右侧全肺切除4例，左侧1例），全肺切除16例（右全肺7例，左全肺9例），单纯肺叶切除8例，9例患者行肺叶袖式切除术，2例行病灶单纯切除，术后病理报告均为腺样囊行癌。清扫肺门及纵隔淋巴结4例见癌转移，支气管残端癌残留5例。3例在纤支镜下介入治疗，（其中1例肿瘤位于气管下段后壁，1例位于左总支气管，另1例位于右上叶支气管前段）。术后21例患者辅助放疗（含5例残端阳性者及4例有淋巴转移患者），放射总剂量分别为60 Gy-100 Gy。5例化疗（含3例纤支镜下介入治疗患者），另17例未作任何辅助治疗。

## 2 结果

全组43例患者术后均无并发症发生，无手术死亡。总随访率为97.6%（42/43），1例失访按死亡计。3年、5年、10年生存率分别为100%（41/41）、89.5%（34/38）、87.1%（27/31）。术后辅助放疗者5年生存率为100%（21/21）。5例气管残端阳性者手术后经放疗均获得长期生存。17例未做任何辅助治疗者有2例死于复发和转移。内镜介入治疗的3例患者中1例于术后6年出现双肺转移，该患者因合并慢性阻塞性肺疾病10余年，最后死于肿瘤消耗合并呼吸衰竭。术后残端阳性与阴性患者的5年生存率分别为100%（4/4）*vs* 88.23%（30/34），*P*>0.05；术后辅助化、放疗与未做辅助治疗患者的5年生存率分别为8.33%（2/24）*vs* 7.14%（1/14），*P*>0.05，差异均无统计学意义。

## 3 讨论

腺样囊性癌是一种少见的气管、支气管内肿瘤，又名圆柱瘤。起源于气管、支气管粘液腺的导管，生长缓慢^[3]^，1982年世界卫生组织发表的肺肿瘤组织分类法将腺样囊性癌和粘液表皮癌归类于支气管腺体癌。

### 3.1 发病情况及病理特点

气管、支气管腺样囊性癌发病率较低^[1, 4]^，本组病例占我们同期收治的支气管肺部肿瘤的0.95%。平均年龄为43岁，低于同期肺癌患者。该病在支气管低度恶性肿瘤中发病率也较低^[5]^，占气管原发肿瘤的30%^[6]^。男女发病率相近^[4]^，本组患者男女比例为1.15:1。腺样囊性癌来源于支气管粘膜的腺管或腺体的粘液分泌细胞，多发生在第6级支气管以上、较中心有粘液腺结构的气管、支气管内，有文献报道^[7]^气管后侧软骨和膜部的连接部分为肿瘤的好发部位，与本组病例统计结果不一致。此瘤发生在粘膜下，常可在粘膜下平面围绕着支气管壁蔓延较远的距离，其生长方式为浸润性生长，瘤体外无完整包膜。有学者^[8]^把气管腺样囊性癌根据镜下细胞排列分为三个亚型：腺管状、筛状、实体状。其中筛状是指肿瘤细胞由位于内层的导管上皮细胞和外层的肌上皮细胞组成，两种细胞排列呈典型筛状，筛孔内有粘液样物。组织学分级：Ⅰ型包含腺管状和筛状，无实体状；Ⅱ型指腺管状、筛状和包含 < 20%的实体状；Ⅲ型指腺管状、筛状和>20%的实体状。浸润类型：Ⅰ型完全在腔内；Ⅱ型主要在腔内；Ⅲ型主要在腔外，分化最好的是管状，最差的是实体状。组织学亚型是该病肿瘤生长方式和预后的重要因素。按Bhattaacharwa^[9]^在2004年SEER总体分析中的建议，病理分期可分为以下几种：T1：气管肿瘤 < 2 cm；T2：气管肿瘤>2 cm；T3：肿瘤侵及气管外，但未侵及邻近器官或组织；T4：肿瘤侵及邻近器官或组织；Tx：肿瘤无法评估。颈部或纵隔淋巴结阳性为N1。Ⅰ期为T1N0；Ⅱ期为T2N0；Ⅲ期为T3N0；Ⅳ期为T4N0或任何TN1。

### 3.2 影像学表现

气管腺样囊性癌有不同的生长方式及影像表现，有文献^[10]^报道根据其影像表现分为腔内广基型、管壁浸润型、腔内外生长型及隆突肿块型等四型。气管体层摄影、胸部CT及MRI均可显示肿瘤。气管体层摄影能够比较清楚地显示肿瘤长度、管腔内外肿瘤及管壁情况，而CT则能更清楚显示腔内特别是腔外受侵的范围与淋巴结转移的情况，增强扫描病灶呈不同程度均匀强化。MRI较CT能更清楚地分辨肿瘤的密度。本组15例为仅行气管体层检查而发现原发肿瘤，另28例行CT检查，其中7例行MRI检查。其表现与文献报道基本一致，鉴于如今高分辨螺旋CT在临床的广泛应用，我们认为目前对初诊的腺样囊性癌患者行胸部CT并配合纤支镜检查即可，必要时再行胸部MRI，气管体层摄影可不作为常规检查。

### 3.3 临床特点

腺样囊性癌的病史2月-10余年，多在2年-3年，本组最长者为4年。早期肿瘤较小，一般无临床症状，偶有刺激性咳嗽伴血丝痰，病情进展缓慢，持续时间较长，咯血量一般很少，本组有7例患者仅为痰中少量带血。当肿瘤增大至影响通气时，可出现反复呼吸道感染，甚至出现肺不张、肺脓肿。当患者出现呼吸道不全梗阻时，最常见的症状是呼吸困难和喘鸣，易误诊为慢性支气管炎或支气管哮喘。本组有6例患者因肿瘤引起气道不全梗阻致喘息伴吸气性哮鸣音而就诊。腺样囊性癌好发于主、叶支气管，较小支气管少见，如为周围型，则表现为单发圆形或类圆形阴影。张合林报道^[5]^8例患者中有2例为周围型，本组周围型发生率为6.9%（3/43）。

### 3.4 治疗情况及预后

目前认为根治性手术切除是气管、支气管腺样囊性癌的首选治疗方法，但由于肿瘤沿着管壁浸润，显微镜下观察肿瘤总是超过可见或可触及的肿瘤界限，这种扩散在很多病例达1 cm或更多。故手术切缘阳性较多见。本组40例手术患者，单纯全肺切除16例，行气管成型者14例，仍有5例残端阳性。气管腺样囊性癌对放疗较敏感，Kanematsu等^[11]^报道16例腺样囊性癌患者，其中11例接受手术治疗，6例为切缘阳性，5例阳性患者在术后进行放疗，其5、10年无复发生存率分别为91%和76%。另有文献^[12]^报道，对不能手术的患者采用同步放疗、化疗，提示取得较好的结果。本组5例残端阳性患者术后配合放疗，目前均获得长期生存。本组患者中3例未手术，在内镜下高频圈套切除并配合冷冻治疗，术后化疗，1例6年后死亡，另2例目前生存状况良好。有文献^[13]^报道，经纤支镜腔内激光、微波、高频电刀切割、支架植入等方法作为姑息性治疗手段，可有效切除肿瘤并缓解呼吸道症状。

综上所述，我们认为对长期伴有呼吸道症状的患者应提高警惕，必要时行纤支镜及胸部CT检查，避免遗漏腺样囊性癌患者，确诊者首选手术治疗并配合放疗，不能手术者在内镜下介入治疗也是一种较好的治疗方法。
